# Comparative Evaluation of Select Serological Assays for Zika Virus Using Blinded Reference Panels

**DOI:** 10.3390/v16071075

**Published:** 2024-07-03

**Authors:** Devy M. Emperador, Mars Stone, Eduard Grebe, Camille Escadafal, Honey Dave, Eve Lackritz, Cassandra Kelly-Cirino, Ingrid Rabe, Diana P. Rojas, Michael P. Busch, Graham Simmons

**Affiliations:** 1 Pandemic Threats Programme, Foundation for Innovative New Diagnostics (FIND), 1218 Geneva, Switzerland; devy.emperador@finddx.org (D.M.E.);; 2 Vitalant Research Institute, San Francisco, CA 94105, USA; 3 Department of Laboratory Medicine, University of California San Francisco, San Francisco, CA 94143, USA; 4 Epidemic and Pandemic Preparedness and Prevention Department, Health Emergencies Programme, World Health Organization, 1211 Geneva, Switzerland

**Keywords:** Zika virus, dengue virus, IgM, IgG, diagnostics

## Abstract

In response to the 2015 Zika virus (ZIKV) epidemic that occurred in Brazil, numerous commercial serological assays have been developed for clinical and research applications. Diagnosis of recent infection in pregnant women remains challenging. Having standardized, comparative studies of ZIKV tests is important for implementing optimal diagnostic testing and disease surveillance. This is especially important for serology tests used to detect ZIKV infection given that antibodies against ZIKV can cross-react with other arboviruses in the same virus family, such as dengue virus (DENV), yellow fever virus (YFV) and West Nile virus (WNV). We looked at the sensitivity and specificity of tests detecting ZIKV antibodies (IgM, IgG) from multiple manufacturers using panels of samples previously collected with known exposure to ZIKV and other arboviruses. We found that performance of the IgM tests was highly variable, with only one test (Inbios 2.0 IgM capture ELISA) having both high sensitivity and specificity. All IgG tests showed good sensitivity; however, specificity was highly variable, with some assays giving false-positive results on samples infected by another flavivirus. Overall, the results confirmed that accurate ZIKV antibody testing is challenging, especially in specimens from regions endemic for multiple other flaviviruses, and highlight the importance of available and suitable reference samples to evaluate ZIKV diagnostics.

## 1. Introduction

The Zika virus (ZIKV) outbreaks in the Western Pacific and the Americas from 2013 to 2017 demonstrated a causal link between ZIKV infection and neurological complications including congenital Zika syndrome (CZS) and other fetal neurodevelopmental disorders, among a subset of infections during pregnancy, and Guillain-Barré syndrome in infected adults [1]. Following infection, viral RNA is generally only detectable up to 10 days post-symptom-onset in serum and has often waned by the time symptoms occur, limiting the utility of molecular testing among pregnant women who either do not present early enough for RNA detection or who are asymptomatic. More recent research suggests that viral RNA persists in some compartments beyond 10 days but not in all infected persons [2].

Immunoglobulin M (IgM) is generally detectable from the second week post-symptom-onset onward, followed within days by IgG [3,4]. While molecular detection is the preferred diagnostic method for ZIKV, given the brevity of RNA detection window, the assessment of the performances of IgM assays is critical to determine their utility in identifying recent infection and guiding pregnancy monitoring, particularly as many infections are asymptomatic [5]. In regions with no known active ZIKV transmission, IgM detection could allow for the detection of ZIKV in the general public and help to track re-emergence and risk in women of reproductive age. Furthermore, IgG assay performance has been less commonly explored for acute diagnostic purposes but may be of interest in combination with IgM and for the determination of population seroprevalence.

At the start of the ZIKV outbreak in the Americas in 2015, diagnostic tests were limited to those developed by a small number of laboratories and hindered by a lack of reference specimen availability; no commercial ZIKV assays were available. Within the first few months of 2016, emergency use assessment and listing (EUAL) procedures were granted to a small number of molecular assays by the WHO [6], while several emergency use authorizations (EUA) were granted for molecular and serologic assays by the U.S. Food and Drug Administration (FDA) [7]. The market for available tests then expanded dramatically, with many commercial assays available beyond those with WHO EUAL or FDA EUA. However, serological tests continued to be plagued with cross-reactivity [8], non-specific reactivity, and prolonged duration of IgM detection [9], which are of particular concern for determining the timing and duration of infection with ZIKV relative to pregnancy. Given these challenges, there continues to be a need to evaluate tests for use in clinical decision-making for ZIKV disease to prepare for future ZIKV epidemics. While WHO international standards have been developed to assess molecular and neutralizing antibody tests [9,10], there are few ZIKV IgM and IgG antibody standards.

The lack of access to archived clinical samples and the decline in ZIKV cases after 2016 necessitates the use of reference panels from existing collections to evaluate and compare the performances and validate the diagnostic accuracy levels of ZIKV serological assays. Vitalant Research Institute (VRI) has conducted multiple studies of ZIKV, dengue viruses (DENVs), and West Nile virus (WNV) based on large-scale screening of blood donors in the US, including Puerto Rico. Viral RNA and virus-specific antibody testing was conducted on the initial donated blood products, and specimens collected during the prospective follow-up of infected donors were tested. These projects have included limited evaluations of the performances of molecular and serological assays and, importantly, established repositories to support future research on and advancement of testing technologies.

The availability and use of reference panels enables and facilitates a more standardized approach to conducting external diagnostic assay performance studies, especially during inter-epidemic periods. Building off the ongoing work by VRI to evaluate the performance of blood donor screening and diagnostic assays, here, we describe a comparative performance evaluation of commercially available diagnostic assays for the detection of ZIKV antibodies using a pre-defined reference panel and provide results from the evaluation to inform the use of these assays for ZIKV serodiagnosis.

## 2. Materials and Methods

### 2.1. Test Selection

A call for partners, managed by the Foundation for Innovative New Diagnostics (FIND), was conducted in October 2019 for ZIKV test manufacturers interested in a head-to-head performance evaluation of their assays. Submissions for inclusion in the head-to-head study were evaluated based on the following criteria: (1) the assay must be design-locked and commercially available; (2) the manufacturer must have a quality management system to manufacture tests in place (e.g., ISO certification, good manufacturing practice); (3) the assay must be approved for use by a regulatory body or have WHO EUAL/FDA EUA. Assays meeting these criteria were tentatively included in the evaluation. Manufacturers who accepted inclusion of their product in the study signed a material transfer agreement to receive panels for the evaluation.

### 2.2. Study Design

This was a multi-laboratory evaluation study of selected ZIKV serological diagnostic tests using blinded panels of well-characterized samples to verify diagnostic test accuracy. The primary objective was to determine diagnostic accuracy for ZIKV IgM or IgG detection against a panel of samples that included ZIKV and other viruses in the flavivirus family (i.e., dengue virus [DENV] and West Nile virus [WNV]), as well as the alphavirus, chikungunya virus [CHIKV] that were antibody-positive and known to be noninfected. Sensitivity analysis panels were constructed using well-characterized seropositive longitudinal samples selected from blood donors confirmed to be ZIKV RNA-reactive at index donation and then enrolled in a follow-up study [2]. Secondary objectives included the assessment of performance in terms of the detection of seroconversion and evaluation of the length of the seropositivity detection period. Thus, in addition to confirmed seropositive samples included in the sensitivity panel, ZIKV RNA-reactive samples from the same longitudinal blood donors that tested negative on routine serological screening assays (for example, the CDC IgM antigen capture assay) were also included as indications of potential enhanced performance for the detection of early seroconversion or delayed seroreversion.

### 2.3. Sample Selection, Reference Tests, and Panel Development

Reference panels were designed using plasma obtained from de-identified blood donor samples from repositories maintained at VRI. ZIKV RNA-reactive blood donations and subsequent samples from blood donors enrolled in follow-up studies were obtained from twelve donors in Puerto Rico or the continental US in 2016 and 2017 [2,11]. DENV RNA-reactive blood donations and follow-up samples were collected in Puerto Rico in 2010–2013 [12], when DENV-1 and -4 were the predominant circulating serotypes [13] Based on ages of blood donors and DENV IgG status at time of RNA-reactive index donation, it is assumed that all cases were secondary infections. WNV RNA-reactive blood donations and follow-up samples were collected in the continental US in 2005 and 2006 [14]. Cross-sectional blood donations from before, during, and after the Puerto Rico CHIKV epidemic in 2014 were also included [15]. These samples were all seronegative for DENV IgM, consistent with the finding that there was little circulating DENV that year [15]. All but one was DENV IgG-positive, as expected from samples collected in a DENV endemic region. However, the lack of IgM and circulating DENV suggests that these specimens do not represent acute DENV responses. All samples had previous donor consent for the use of their leftover samples for further research purposes. The estimated dates of ZIKV RNA detectable infection were deduced in seronegative index blood donations based on viral load and viral doubling time, as previously described [2,11].

The reference tests used in this study are detailed in Table 1. In brief, the CDC Zika monoplex quantitative real-time reverse transcription polymerase chain reaction (RT-PCR) assay was used as the reference test to confirm ZIKV infection and determine RNA concentrations. A CDC IgM-capture enzyme-linked immunosorbent assay (MAC ELISA, [16]) was used to confirm the presence of ZIKV IgM and confirm previous exposure to ZIKV. Flavivirus-negative and CHIKV-positive samples were collected prior to the ZIKV epidemic. The Bio-Techne IgG ELISA (Minneapolis, MN, USA) was used to establish the presence of ZIKV IgG. Tests for other arbovirus antibodies are described in Table 1.

Each of the IgM and IgG evaluation panels are described in Table 2, and they consisted of ZIKV IgM- (*n* = 40) or IgG (*n* = 41)-positive samples from ZIKV RNA-reactive blood donors and ZIKV IgM- or IgG-negative samples from confirmed ZIKV RNA-negative individuals (*n* = 72). The ZIKV-positive sensitivity panels were collected from twelve ZIKV RNA-reactive blood donors, all of whom were ZIKV IgM-negative using the reference assay (CDC MAC ELISA) at the index donation. For each donor, 3–5 longitudinal time-points representing days 5–180 post-index donation for the IgM panel and days 6–399 for the IgG were used. Of the 72 ZIKV IgM/IgG-negative samples comprising the specificity panels, a large proportion came from flavivirus (DENV or WNV) RNA-reactive blood donors and were confirmed to be seropositive via ELISA. Specimens with a range of signal intensities using the reference IgM or IgG serology were selected. The sample size used to assess diagnostic accuracy was determined based on an expected sensitivity of 95% and specificity of 90% for the diagnostic tests used to detect ZIKV IgM or IgG so that the study would have 80% power to obtain the estimates with a precision of +/−10% [17].

**Figure 1:**
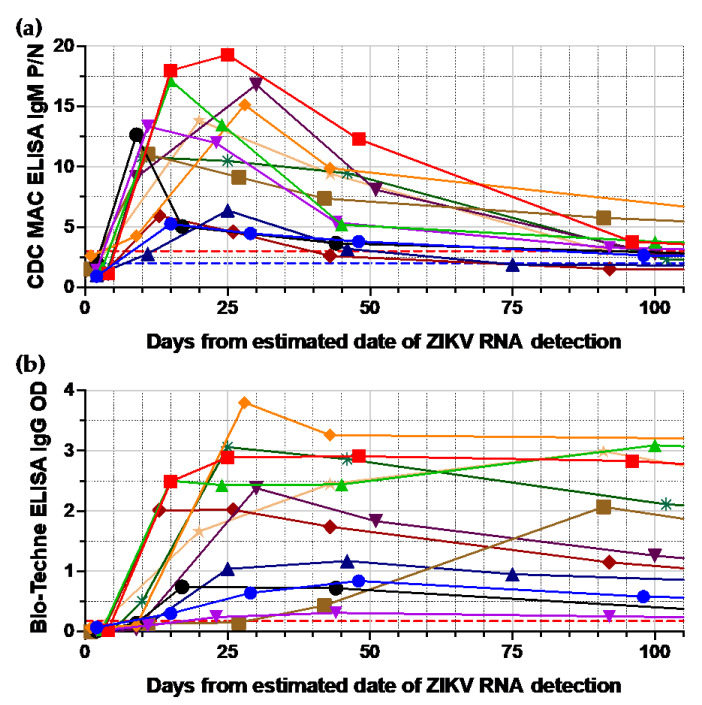
Anti-ZIKV IgM (**a**) and IgG (**b**) signal intensity over time using reference assays. Longitudinal samples from 12 donors were tested using the reference assays for IgM (**a**) and IgG (**b**). The red horizontal line shows the cutoff for a reactive interpretation, and the blue horizontal line shows the cutoff for an equivocal interpretation. Each color line represents a donor (*n* = 12).

An additional set of samples composed of over 20 samples from nucleic acid testing (NAT) confirmed that ZIKV infected blood donors collected before (i.e., prior to seroconversion) and after (i.e., post-seroreversion) the period that IgM or IgG was detectable using the reference assay (Table 2). The addition of this last set of samples was intended to take advantage of longitudinal samples from ZIKV-RNA-reactive donors and allow for the potential identification of tests with a higher sensitivity than the reference tests. Figure 1 shows the longitudinal trajectories of IgG and IgM signal intensity (net optical density [OD] or positive/negative [P/N]) using the reference assays.

### 2.4. Study Conduct

Based on the assay, identical IgM and/or IgG blinded reference panels and reporting templates were sent from VRI to the manufacturer or an external laboratory designated by the manufacturer and tested according to the assay’s instructions for use. Results were returned to VRI using a provided standardized reporting template within 6 months of receipt for unblinding and analysis. Unblinded raw data were subsequently shared with the manufacturer at study completion.

### 2.5. Data Analysis

Primary and subgroup clinical sensitivity and specificity were estimated as the binomial probability of a reactive result and non-reactive result in confirmed-positive and confirmed-negative samples, as determined using the reference assays, respectively. Confidence intervals were computed using the Wilson score method, which is valid for probabilities close to zero and one. All analyses were conducted using the R statistical programming language (version 4.0.4, R Foundation for Statistical Computing 2021 [https://www.r-project.org/], accessed on 16 June 2024), and confidence intervals were computed using the binom package (version 1.1-1, S Dorai-Raj 2014 [http://CRAN.R-project.org/package=binom], accessed on 16 June 2024).

## 3. Results

### 3.1. Test Selection

Fifteen serological tests from five test manufacturers were submitted in response to a call for partners led by FIND. All submissions met inclusion criteria for the head-to-head performance evaluation. Of the five test manufacturers, four agreed to the material transfer agreement to participate in this study. One test manufacturer declined to participate.

A total of 14 tests were included in the evaluation, with assays ranging from traditional ELISAs to rapid diagnostic tests (Table 3). Blinded reference panels were sent to the four test manufacturers, and results were received within 6 months after receipt for 12 tests. The results from two of the tests (STANDARD F Zika IgG/IgM and STANDARD Q ZIKV/DENV/CHIKV Fast Quad) were not available as the manufacturer informed the team that there were no assays available in stock during the evaluation.

### 3.2. Evaluation Study

#### 3.2.1. Overall Assays Performance

The sensitivity of IgM assays was highly variable, ranging from 10.0% (95% CI: 4.0–23.1%) for the EUROIMMUN Anti-ZIKV ELISA to 97.5% (95% CI: 87.1–99.6%) for the SD Biosensor STANDARD assay (Table 4). Four of the seven IgM assays had clinical specificity above 90%. Two of the assays (EUROIMMUN IIFT Arbovirus Fever Mosaic 2 IgM and EUROIMMUN Anti-Zika virus IIFT IgM) had mid-range sensitivity between 60 and 70%. Interestingly, false negatives clustered with particular study participants, regardless of time since infection. For the Arbovirus Fever Mosaic 2 IgM assay, 9 of 13 false negatives were seen in specimens from just 3 out of 12 participants. Moreover, 10 out of 14 false negatives were found in specimens from four participants for the Anti-Zika virus IIFT IgM assay. For one participant, all four time-points were negative in both assays, despite all being positive for the CDC MAC ELISA, STANDARD Q Arbo Panel I, and EUROIMMUN Anti-Zika Virus ELISA IgAM. The InBios ZIKV Detect 2.0 IgM Capture ELISA failed to detect the final time-point of the longitudinal series. Specificity ranged from 56.9% (95% CI: 45.4–67.7%) to 98.6% (95% CI: 92.5–99.8%). Only the InBios ZIKV Detect IgM assay had a clinical sensitivity and clinical specificity above 90% (Table 4).

High test sensitivity, up to 100%, was observed in all five IgG assays (Table 4). However, overall clinical specificity varied, ranging from 45.8% (95% CI: 34.8–57.3%) for the EUROIMMUN Arbovirus Fever Mosaic assay to 95.8% (95% CI: 88.5–98.6%) for the NovaTec NovaLisa. Two of the five IgG tests had specificity greater than 90%.

#### 3.2.2. Cross-Reactivity

Substantial cross-reactivity to samples from blood donors with a history of recent infection by arboviruses other than ZIKV was observed in both IgM and IgG assays, although some assays achieved high specificity in specimens obtained from donors with antibodies following confirmed DENV or WNV infections or CHIKV seropositive samples collected during an epidemic (Table 5). Two of five IgG assays achieved specificity above 80% in samples from RNA-confirmed DENV-seropositive donors, three in RNA-confirmed WNV-seropositive samples and three in CHIKV-seropositive samples. Three of seven IgM assays had specificities above 80% in DENV-positive samples, with most assays generally demonstrating higher specificities on WNV-positive and CHIKV-positive samples (Table 5).

All but the InBios IgG assay detected the presence of IgG in many of the specimens collected less than 7 days from estimated time of ZIKV RNA positivity (Table 6), despite all of these samples being seronegative for both IgG and IgM on the reference tests. All of the assays detected IgG more often in specimens >180 days compared to the reference test (Bio-Techne IgG ELISA).

## 4. Discussion

In this study, we evaluate the performances of seven IgM and five IgG serology tests using a clinical panel of ZIKV RNA-positive, arbovirus-co-infected, and ZIKV-negative samples from ZIKV endemic and non-endemic regions. Understanding the performances of ZIKV IgM assays is key to understanding their use in the diagnostic algorithm. The four IgM tests in the ELISA format demonstrated the highest specificities (>90%) (Table 4), each of them with different degrees of cross-reactivity with positive samples for IgM against DENV, WNV, and CHIKV (Table 5). Only the InBios ZIKV Detect^TM^ 2.0 IgM Capture ELISA demonstrated both high sensitivity and specificity (95.0% and 93.1%, respectively); however, this assay had a moderate specificity (75.0%) among ZIKV IgM-negative/DENV IgM-positive samples. This has been reported in previous studies [18] and can be explained by the fact that the InBios ELISA is the most comparable in its test structure to the reference test, the CDC Zika MAC-ELISA, as it uses an E/prM recombinant protein [18,19]. In contrast, the NovaTec and the EUROIMMUN ELISAs are based on the NS1 protein, which is expected to confer higher test specificity regarding other flaviviruses, most importantly DENV [20]. The sensitivity levels of the EUROIMMUN IgM assays were higher in their IIFT format than in the ELISA format (Table 4), as previously reported [21]. However, lower specificities were observed for the IIFT, especially with respect to cross-reactivity with DENV- and WNV IgM-positive samples (Table 5). Interestingly, among all Zika IgM assays included in the evaluation, which requires specialized equipment and training to conduct, the only rapid test (STANDARD Q ARBO I from SD Biosensor) demonstrated the highest sensitivity (97.5%), although this was at the expense of specificity (56.9%), again performing poorly on DENV-, WNV-, and CHIKV IgM-positive samples (Table 5).

Although the sensitivity of IgM assays is important to increase the detection of IgM-positive samples, a drawback to high sensitivity is the potential detection of persistent IgM beyond a clinically useful period for identifying recent infection. This is of high importance for determining the risk of ZIKV infection in pregnant women, where infection during the first or second trimester significantly increases the risk of microcephaly. In this respect, no IgM assay that demonstrated a reasonable level of sensitivity performed better than the CDC Zika MAC-ELISA (Table 6), although the addition of IgA to the EUROIMMUN IgM ELISA approached a sensitivity similar to that of the CDC MAC-ELISA on early time-points (17/22 and 21/22 seropositives at 7–29 days, respectively) without an increase in inappropriate sensitivity at later time-points.

Regarding the less clinically useful IgG assays, the overall test sensitivity and specificity results demonstrate a high degree of variation in performance except for the clinical sensitivity of the IgG tests, estimated at 95–100% for all assays evaluated. The NovaTec and InBios IgG ELISAs demonstrated the highest specificity (>94%), followed by the EUROIMMUN IgG ELISA (80.6%), with cross-reactivity generally highest against acute DENV samples. As reported previously [21], EUROIMMUN IgG IIFT demonstrated the lowest specificity, with high levels of cross-reactivity with all DENV- and WNV IgG-positive samples (Table 5). Despite its performance, the role for IgG testing remains unclear.

A notable value of the composition of these evaluation panels is the inclusion of RNA-reactive index blood donation samples confirmed via ELISA and neutralization tests for antibodies against three arboviruses (WNV, DENV, CHIKV), as well as the use of highly characterized longitudinal RNA and serologically confirmed ZIKV-positive samples from ZIKV non-endemic and endemic countries. Flavivirus serological assays are prone to high levels of cross-reactivity, particularly in the early convalescent phase of 2 to 12 weeks after infection when the induction of antibodies from memory B cells producing cross-reactive antibodies predominate [22]. These cross-reactive antibodies tend to be transiently produced early after infection and decline over several months, leaving a type-specific response [23,24]. Furthermore, repeated exposure to flavivirus infections increases problems due to cross-reactivity, with non-specific responses to ZIKV becoming more pronounced after secondary, compared to primary, DENV infections [25]. Therefore, the analysis of flavivirus samples collected in endemic regions and from the early convalescent stage is critical for the characterization of the specificity of assays. For example, on longitudinal samples from ZIKV RNA-reactive donors, many of the IgG assays were positive at early time-points (Table 6), even prior to IgM seroconversion. This was true even for assays such as the NovaTec NovaLisa^®^ Zika Virus IgG ELISA, which demonstrated a high degree of overall specificity (Table 4). It is likely that this is due to the detection of transient cross-reactive anti-DENV memory responses being boosted by acute ZIKV infection. While this cross-reactivity is not ideal, in some settings, such as a ZIKV epidemic in a situation where there is no co-circulating DENV, the inadvertent enhanced sensitivity may be beneficial; however, this must be differentiated from secondary dengue virus infection. Some assays, such as the NovaTex IgM, returned a false-positive result on one out of six samples from recent CHIKV-infected individuals. While cross-reactivity with alphaviruses is less common, it has been reported previously [26].

One notable strength of this study was that separate evaluation panels were sent for the evaluation of multiple tests from each manufacturer, with the samples in each panel coded with unique identifying bar codes. This allowed for complete blinding within laboratories evaluating multiple tests such that comparison between the results of two tests on a same sample was not possible. Similarly, the available panels included longitudinal samples to allow the assessment of performance, which provides a better assessment of the use of IgG and IgM assays within a ZIKV diagnostic algorithm.

There are several limitations to this study. First, there was a lower-than-anticipated number of positive responses to the call for commercial test manufacturer partners launched compared to the number of tests available commercially at the time of the study. In the future, more frequent calls for partners could allow for increased participation, particularly after this first call. Similarly, as several evaluation panels are still available at VRI, the Zika diagnostic community could identify ZIKV serological tests of most interest that were not included in this evaluation and allow us to conduct a second round of evaluations using these available panels, either by the test manufacturer or at an independent laboratory.

Another limitation was that the evaluation panels were sent for testing to either the test manufacturer or a laboratory designated by the test manufacturer for the evaluation, allowing for tests to be performed by experienced staff in an ideal environment. Given the time and resources that would have been required to train an independent laboratory to conduct all tests being evaluated, the provision of a blinded panel to the manufacturer labs allowed for evaluations to be conducted in a timelier manner. However, there is still a need to understand diagnostic accuracy among laboratories in different settings, particularly in endemic-country laboratories that will be conducting routine ZIKV testing.

Other limitations are linked to the compositions of the evaluation panels. False negatives reported by two of the IgM assays clustered to particular participants in the sensitivity panel, highlighting a potential flaw to using longitudinal samples to calculate sensitivity and specificity, but also demonstrating the potential structural limitations of some assays. Since the samples in the panels originated from infected blood donors identified in North America (mainly Puerto Rico), the results from this evaluation may not be indicative of the test performance observed in other regions of the world with circulation of differing flaviviruses. Similarly, the current panels did not include patient samples positive for antibodies against other important arbovirus circulating in the Americas such as Mayaro virus and yellow fever virus; for yellow fever virus in particular, it is important to assess levels of cross-reactivity due to wild-type infection and vaccination. In the future, it would be important to work with different partners to develop new evaluation panels with clinical samples from other geographical areas (South America, Asia and Africa) including positive samples for antibodies against other arboviruses circulating in these regions.

Diagnostic test evaluations and the availability of suitable reference panels have been highlighted as key activities under the WHO R&D Blueprint Roadmap for ZIKV [27]. As more diagnostic tests for arboviruses continue to be developed and become commercially available, questions on the potential role of serological tests in the early detection and differentiation of arbovirus diseases will remain, requiring the availability of suitable reference materials for verification in the laboratory, as well as allowing for more easier comparison of performance among different tests.

## Figures and Tables

**Table 1:** Description of reference tests for sample panels.

Virus	IgM Detection	IgG Detection
Zika	MAC-ELISA (US CDC)	ELISA (Bio-Techne, Minneapolis, MN, USA)
Dengue	ELISA (Focus Diagnostics, San Juan Capistrano, CA, USA) and/or ELISA (InBios International, Seattle, WA, USA)	ELISA (Focus Diagnostics, San Juan Capistrano, CA, USA)
Chikungunya	ELISA (EUROIMMUN AG, Lübeck, Germany)	ELISA (EUROIMMUN AG, Lübeck, Germany)
West Nile	ELISA (Focus Diagnostics, San Juan Capistrano, CA, USA)	ELISA (Focus Diagnostics, San Juan Capistrano, CA, USA)

**Table 2:** Clinical evaluation panel for ZIKV serological assays.

Group	Subgroup	*n* Samples
**IgM Evaluation Panel**		112
Zika IgM-positive		40
Zika IgM-negative		72
ZIKV/DENV non-endemic region (US)	Healthy blood donors	24
	WNV IgM-positive *	10
Arbovirus endemic region (Puerto Rico)	ZIKV-, DENV-, CHIKV IgM-negative *	12
	DENV IgM-positive *	20
	CHIKV IgM-positive *	6
**IgG Evaluation Panel**		113
Zika IgG-positive		41
Zika IgG-negative		72
ZIKV/DENV non-endemic region (US)	Healthy blood donors	24
	WNV IgG-positive *	10
Arbovirus endemic region (Puerto Rico)	ZIKV-, DENV-, CHIKV IgG-negative *	12
	DENV IgG-positive *	20
	CHIKV IgG-positive *	6
**Longitudinal Panels**		
ZIKV RNA-positive, collected before and after the period of detectable IgM		26
ZIKV RNA-positive, collected before and after the period of detectable IgG		23

* Samples collected prior to the 2015 ZIKV epidemic.

**Table 3:** ZIKV tests included in the head-to-head evaluation.

Test Name	Test Manufacturer, Location	Test Type	Test Antigen (ZIKV)	Test Target (ZIKV)	Multiplex (Y/N)	Regulatory Status
Anti-Zika Virus ELISA IgAM	EUROIMMUN AG, Lübeck, Germany	ELISA	NS1	IgA, IgM	N	CE-IVD
Anti-Zika Virus ELISA IgM	EUROIMMUN AG, Lübeck, Germany	ELISA	NS1	IgM	N	CE-IVD
Anti-Zika Virus ELISA IgG	EUROIMMUN AG, Lübeck, Germany	ELISA	NS1	IgG	N	CE-IVD
Anti-Zika virus IIFT IgG	EUROIMMUN AG, Lübeck, Germany	IIFT	Infected cells	IgG	N	CE-IVD
Anti-Zika Virus IIFT IgM	EUROIMMUN AG, Lübeck, Germany	IIFT	Infected cells	IgM	N	CE-IVD
IIFT Arbovirus Fever Mosaic 2 IgG	EUROIMMUN AG, Lübeck, Germany	IIFT	Infected cells	IgG	Y	CE-IVD
IIFT Arbovirus Fever Mosaic 2 IgM	EUROIMMUN AG, Lübeck, Germany	IIFT	Infected cells	IgM	Y	CE-IVD
ZIKV Detect 2.0 IgM Capture ELISA	InBios International, Inc., Seattle, WA, USA	ELISA	E/prM	IgM	N	CE-IVD, US FDA De Novo
NuGen ZIKV IgG Capture ELISA	InBios International, Inc., Seattle, WA, USA	ELISA	NS1	IgG	N	RUO
NovaLisa Zika Virus IgM μ-capture ELISA	NovaTec Immunodiagnostica GmbH, Dietzenbach, Germany	ELISA	NS1	IgM	N	CE-IVD
NovaLisa Zika Virus IgG ELISA	NovaTec Immunodiagnostica GmbH, Dietzenbach, Germany	ELISA	NS1	IgG	N	CE-IVD
STANDARD Q Arbo Panel I	SD Biosensor, Suwon, Republic of Korea	Lateral flow RDT	-	IgM	Y	CE-IVD

Abbreviations: CE-IVD: European Conformity for In Vitro Diagnostics. ELISA: enzyme-linked immunosorbent assay, IIFT: indirect immunofluorescence test, RDT: rapid diagnostic test, RUO: research use only, FDA: Food and Drug Administration.

**Table 4:** Test sensitivity and specificity of ZIKV IgM and IgG serological assays.

Tests	Sensitivity (95% CI) *, *n*	Specificity (95% CI) *, *n*
**IgM Assays**		
STANDARD Q Arbo Panel I	97.5% (87.1%, 99.6%), 40	56.9% (45.4%, 67.7%), 72
InBios ZIKV Detect 2.0 IgM Capture ELISA	95.0% (83.5%, 98.6%), 40	93.1% (84.8%, 97.0%), 72
EUROIMMUN Anti-Zika Virus ELISA IgAM	72.5% (57.2%, 83.9%), 40	93.1% (84.8%, 97.0%), 72
EUROIMMUN IIFT Arbovirus Fever Mosaic 2 IgM	67.5% (52.0%, 79.9%), 40	76.4% (65.4%, 84.7%), 72
EUROIMMUN Anti-Zika virus IIFT IgM	65.0% (49.5%, 77.9%), 40	61.1% (49.6%, 71.5%), 72
NovaTec NovaLisa Zika Virus IgM μ-capture ELISA	20.0% (10.5%, 34.8%), 40	98.6% (92.5%, 99.8%), 72
EUROIMMUN Anti-Zika Virus ELISA IgM	10.0% (4.0%, 23.1%), 40	93.1% (84.8%, 97.0%), 72
**IgG Assays**		
NovaTec NovaLisa Zika Virus IgG ELISA	100.0% (91.4%, 100.0%), 41	95.8% (88.5%, 98.6%), 72
EUROIMMUN Anti-Zika Virus ELISA IgG	100.0% (91.4%, 100.0%), 41	80.6% (70.0%, 88.0%), 72
EUROIMMUN Anti-Zika virus IIFT IgG	100.0% (91.4%, 100.0%), 41	51.4% (40.1%, 62.6%), 72
EUROIMMUN IIFT Arbovirus Fever Mosaic 2 IgG	100.0% (91.4%, 100.0%), 41	45.8% (34.8%, 57.3%), 72
InBios NuGen ZIKV IgG Capture ELISA	95.1% (83.9%, 98.7%), 41	94.4% (86.6%, 97.8%), 72

* Equivocal results counted as non-reactive.

**Table 5:** Specificity (%) of ZIKV assays using samples with evidence of recent DENV, WNV, and CHIKV infection.

Tests	DENV *n* = 20	WNV *n* = 10	CHIKV *n* = 6
**IgM Assays (95% CI)**			
NovaTec NovaLisa Zika Virus IgM μ-capture ELISA	100.0% (83.9%, 100.0%)	100.0% (72.2%, 100.0%)	83.3% (43.6%, 97.0%)
EUROIMMUN Anti-Zika Virus ELISA IgM	90.0% (69.9%, 97.2%)	70.0% (39.7%, 89.2%)	100.0% (61.0%, 100.0%)
EUROIMMUN Anti-Zika Virus ELISA IgAM	85.0% (64.0%, 94.8%)	80.0% (49.0%, 94.3%)	100.0% (61.0%, 100.0%)
InBios ZIKV Detect 2.0 IgM Capture ELISA	75.0% (53.1%, 88.8%)	100.0% (72.2%, 100.0%)	100.0% (61.0%, 100.0%)
EUROIMMUN IIFT Arbovirus Fever Mosaic 2 IgM	60.0% (38.7%, 78.1%)	60.0% (31.3%, 83.2%)	100.0% (61.0%, 100.0%)
EUROIMMUN Anti-Zika virus IIFT IgM	30.0% (14.5%, 51.9%)	50.0% (23.7%, 76.3%)	66.7% (30.0%, 90.3%)
STANDARD Q Arbo Panel I	20.0% (8.1%, 41.6%)	30.0% (10.8%, 60.3%)	16.7% (3.0%, 56.4%)
**IgG Assays (95% CI)**			
NovaTec NovaLisa Zika Virus IgG ELISA	85.0% (64.0%, 94.8%)	100.0% (72.2%, 100.0%)	100.0% (61.0%, 100.0%)
InBios NuGen ZIKV IgG Capture ELISA	85.0% (64.0%, 94.8%)	100.0% (72.2%, 100.0%)	83.3% ^a^ (43.6%, 97.0%)
EUROIMMUN Anti-Zika Virus ELISA IgG	45.0% (25.8%, 65.8%)	100.0% (72.2%, 100.0%)	83.3% ^a^ (43.6%, 97.0%)
EUROIMMUN Anti-Zika virus IIFT IgG	0.0% (0.0%, 16.1%)	20.0% (5.7%, 51.0%)	0.0% ^a^ (0.0%, 39.0%)
EUROIMMUN IIFT Arbovirus Fever Mosaic 2 IgG	0.0% (0.0%, 16.1%)	0.0% (0.0%, 27.8%)	0.0% ^a^ (0.0%, 39.0%)

For multiplex assays, the ZIKV component only was reported. Abbreviations: DENV—dengue virus; CHIKV—chikungunya virus; WNV—West Nile virus. ^a^ = In total, 5 out of 6 samples with evidence of recent CHIKV infection were also DENV IgG-positive (6/6 were DENV IgM-negative). Little to no DENV circulated in the year of collection, suggesting distant DENV infection.

**Table 6:** Detection of ZIKV IgM and IgG by time from estimated date of ZIKV RNA detection *.

IgM Assay	<7 Days *n* = 12	7–29 Days *n* = 22	30–89 Days *n* = 14	90–179 Days *n* = 10	≥180 Days *n* = 8
CDC MAC ELISA ^a^	0/12 (0%)	21/22 (95%)	12/14 (86%)	5/10 (50%)	1/8 (13%)
EUROIMMUN Anti-Zika Virus ELISA IgM	0/12 (0%)	2/22 (9%)	2/14 (14%)	0/10 (0%)	0/8 (0%)
EUROIMMUN Anti-Zika virus IIFT IgM	1/12 (8%)	16/22 (73%)	9/14 (64%)	2/10 (20%)	3/8 (38%)
EUROIMMUN IIFT Arbovirus Fever Mosaic 2 IgM	3/12 (25%)	17/22 (77%)	10/14 (71%)	3/10 (30%)	6/8 (75%)
EUROIMMUN Anti-Zika Virus ELISA IgAM	0/12 (0%)	17/22 (77%)	12/14 (86%)	3/10 (30%)	0/8 (0%)
NovaTec NovaLisa Zika Virus IgM μ-capture ELISA	0/12 (0%)	4/22 (18%)	4/14 (29%)	1/10 (10%)	0/8 (0%)
InBios ZIKV Detect 2.0 IgM Capture ELISA	0/12 (0%)	22/22 (100%)	13/14 (93%)	8/10 (80%)	6/8 (75%)
STANDARD Q Arbo Panel I	2/12 (17%)	22/22 (100%)	13/14 (93%)	10/10 (100%)	7/8 (88%)
**IgG Assay**	**<7 Days** ***n*** **= 12**	**7–29 Days** ***n*** **= 21**	**30–89 Days** ***n*** **= 12**	**90–179 Days** ***n*** **= 8**	**≥180 Days** ***n*** **= 11**
Bio-Techne Anti-ZIKV IgG ELISA ^a^	0/12 (0%)	14/21 (67%)	12/12 (100%)	8/8 (100%)	7/11 (64%)
EUROIMMUN Anti-Zika Virus ELISA IgG	5/12 (42%)	20/21 (95%)	12/12 (100%)	8/8 (100%)	11/11 (100%)
EUROIMMUN Anti-Zika virus IIFT IgG	10/12 (83%)	21/21 (100%)	12/12 (100%)	8/8 (100%)	11/11 (100%)
EUROIMMUN IIFT Arbovirus Fever Mosaic 2 IgG	10/12 (83%)	21/21 (100%)	12/12 (100%)	8/8 (100%)	11/11 (100%)
NovaTec NovaLisa Zika Virus IgG ELISA	11/12 (92%)	19/21 (90%)	12/12 (100%)	8/8 (100%)	11/11 (100%)
InBios NuGen ZIKV IgG Capture ELISA	0/12 (0%)	18/21 (86%)	12/12 (100%)	7/8 (64%)	11/11 (100%)

* Equivocal results were counted as non-reactive. ^a^ Reference test.

## Data Availability

All relevant data are available at https://doi.org/10.5281/zenodo.12734861 (accessed on 16 June 2024).
